# Effects of Fatigue Damage on the Microscopic Modulus of Cortical Bone Using Nanoindentation

**DOI:** 10.3390/ma14123252

**Published:** 2021-06-12

**Authors:** Xianjia Meng, Chuanyong Qu, Donghui Fu, Chuan Qu

**Affiliations:** Department of Mechanics, School of Mechanical Engineering, Tianjin University, Tianjin 300350, China; xianjiameng@tju.edu.cn (X.M.); testfu@tju.edu.cn (D.F.); quchuan@tju.edu.cn (C.Q.)

**Keywords:** bone fatigue damage, nanoindentation, reduced modulus, fatigue test methods, hierarchical structure

## Abstract

Alterations to the bone structure from cycle loadings can undermine its damage resistance at multiple scales. The accumulation of fatigue damage in a bone is commonly characterized by the reduction in the elastic modulus. In this study, nano-indentation was used for investigating microscopic damage evolution of bovine tibia samples subjected to fatigue loading. Indentation tests were conducted in the same 60 μm × 120 μm area with different degrees of damage, including fracture, and the evolution of reduced modulus was observed. The results showed that bone’s reduced modulus decreased significantly during the initial 40% of the life fraction, whereas it proceeded slowly during the remaining period. As the size of the residual indentations was about 4 μm in length, the degradation of bone’s reduced modulus reflected the accumulation of fatigue damage at smaller scales.

## 1. Introduction

With the aging population, bone health is becoming an increasingly challenging social issue [1,2]. By the year 2025, the economic burden of bone fractures in the United States is estimated to reach $28.5 billion [3]. Clinically, bone mineral density(BMD) is commonly used to evaluate bone quality [4,5]. However, since BMD provides an incomplete picture of bone health, the fatigue damage behavior of bone has been investigated extensively [6,7,8,9,10,11,12].

Bones are natural composite materials with multiscale structures [13,14]. Bone components include collagen and hydroxyapatite, type I collagen fibers, thick and thin lamellar, osteons, and bone matrix, whose size scale could be from the nanoscale to submicroscale [15]. Osteons are the main structural unit of the cortical bone [16,17]. Each osteon is a hollow cylinder with a diameter of approximately 150~300 μm [18,19]. Alterations in bone architecture from fatigue damage can undermine its ability to resist fracture at different spatial scales and eventually cause catastrophic failure [8,20,21]. Studies on bone mechanical behaviors at the macro-scale have been conducted consistently since the 1950s [22,23]. In particular, secant modulus, strain mode, as well as strength and toughness under various loading modes were the major topics studied [6,11,24]. Later, technological advances made it possible to visualize the microscopic structure of bone, allowing for microdamage to be observed by various methods (e.g., light microscope, laser scanning confocal microscope, electron microscope and etc.) [25,26,27,28]. 

Linear microcracks and diffuse damage are the two main types of microdamage that have attracted much attention [23,26,28]. Burr et al. found that linear microcracks (larger than 100 μm) were almost absent in bones until the elastic modulus had decreased by 15% [29]. Before that, fatigue damage appeared on much smaller scale, such as fibrillar sliding and molecular uncoiling [24]. The hierarchical structure of bone determines its excellent strength and toughness [30]. Bone toughness is mostly generated at lager scales (10 μm~1 mm) when bone strength derives from the nano to submicrometer structure [24,31,32]. Detailed studies of the micromechanical properties of bone during fatigue damage progress will contribute to a better understanding of bone strength and may help to explore the conflict between strength and toughness [24,33,34]. As bone remodeling is associated with microdamage, these studies help to explain the relation between bones’ biological adaption capabilities and their micromechanical behavior [35,36,37]. Nanoindentation, avoiding the effects of porosity and damage at larger scale, can be used for investigating the mechanical properties of bone microstructure [38,39,40,41]. Researchers found that the mechanical properties of bone materials varied at osteon, bone matrix and bone trabecular, as well as differences in the thick or thin lamellar of the same osteon [42,43]. Cycle loading affects the damage resistance of bone from nanoscale to macroscale [44]. However, the course of the degradation of the microscopic modulus of compact bone during fatigue loading has not been explored comprehensively before. 

The main strength of this study was to investigate the evolution of bone’s micromechanical properties with fatigue damage. The micro-mechanical properties of the same bone region were tested by nanoindentation. These tests were conducted at different degrees of damage, including fracture. Due to the assumptions of this method and the complex structures of the bone, the extracted values reflect the relative mechanical properties and allow for relative differences to be accurately compared [38,45]. The fatigue induced deterioration of micromechanical properties that cannot be captured by bulk measurements can be determined by the nanoindentation method, including fracture [46,47]. Compared to conventional methods that are used as bone mineral density, macroscopic Young’s modulus, and histological methods, nanoindentation provides a new perspective for evaluating bone fatigue damage.

## 2. Materials and Methods

### 2.1. Specimen Preparation and Fatigue Experiment 

The bovine tibia used in this research was obtained from a slaughtering factory in Tianjin, China. Fresh bovine tibias were dehydrated and dried in air for approximately 7 days before measurement. Thereafter, the osteoepiphysis was removed, and the backbone section that had a radial thickness of approximately 8–10 mm was kept. The specimen that was located perpendicular to the inner and outer surfaces along the axial direction was cut out. This was performed at the periphery of the cross-section, where the curvature was smaller than other parts. This was used as a blank specimen. First, the blank specimen was ground to a dimension of 4.5 mm × 4.5 mm × 32 mm using silicon carbide (SiC) papers that had grits of 600 and 800. Thereafter, the specimen was finely reduced into 4.0 ± 0.15 mm × 4.0 ± 0.15 mm × 30 ± 0.15 mm with SiC papers that had grits pf 1500 and 3000. This was conducted using a P-1 sample polisher (Hong Kong, China). Finally, the surface was polished using SiC papers that had grits of 5000 and 7000. This process was conducted using a precision grinding machine (UNIPOL-802, Tokyo, Japan). Cooling water was continuously poured on the samples throughout the preparation process. Special attention was paid to obtain the surfaces of each sample as parallel as possible. There was no evidence of metabolic bone disease under the optical microscope. Nine dry bone samples were made from the bovine tibia of two individual. 

**Figure 1:**
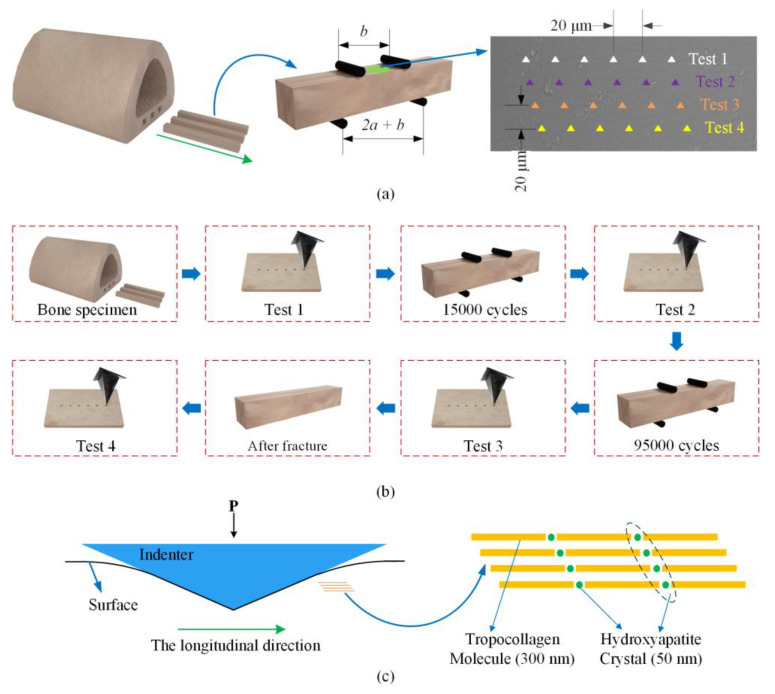
A graphic of the fatigue experiment and nanoindentation test. (**a**) 4-piont bending and the indentation position on the bone. The inner span (*b*) was 10 mm and the outer span (*b* + 2*a*) was 20 mm. (**b**) The experimental process. (**c**) Nanoindentation cross section.

The transverse loading will be present in three-point bending tests and it is difficult to separate the damage caused by fatigue loading from the stress concentration. Thus, four-point bending (Figure 1a) was used in this study so that the region between the central loading points was under constant bending moment. A technique for pure bending of bone samples was applied in this study [48]. The contact radius was 2.0 mm, which was sufficient to avoid stress concentration. Fatigue tests were performed using Electro-Pulsinstron (E10000N, High Wycombe, UK). The rage of the instron was 10 kN and its accuracy was 0.001 N. Three samples were tested to failure without cycling. Stress control was conducted and the maximum stress *σ_xx_* was 120 MPa, where the measured ultimate strength of the samples (*σ_b_*) was 170 ± 8 MPa and *σ_xx_* = 0.7*σ_b_*. The minimum stress in the cycle was 12 MPa. The thickness of the sample is *d*, the width of that is *w* and *F* = *σ_xx_·wd*^2^/3*a*. The type of the loading was a sinusoidal wave and the frequency was 10 Hz. Six samples were tested in the fatigue loading and four of them were subjected to three times cycle tests. Thereby, 15,000 cycles were carried out firstly and then 80,000 cycles were carried out. Finally, all samples were fatigue loaded to fracture. The left two samples failed after 39,000 and 49,000 cycles, and thus underwent only two times cycle loadings. 

### 2.2. Nanoindentation Test

The NANO G200 (produced by the American MTS company, Oak Ridge, TN, USA) was used to perform the nanoindentation tests. The cube indenter has a probe tip end radius less than 20 nm. The surfaces were evaluated using the FM-Nanoview 1000AFM (Frequency: 50 Hz/60 Hz, Beijing, China). During the whole fatigue experiment, the root mean square surface roughness was always less than 60 nm on a calibrated 20 × 20 μm scan [49]. Further, 1000 nm was selected as the ultimate indentation depth. The following indentation activities were performed: (I) loading at a rate of 10 nm/s until the depth reached 1000 nm (resulting in residual indents of about 4 μm in width); (II) holding for 10 s on the peak load; and (III) unloading at a rate of 100 nm/s.

**Figure 2:**
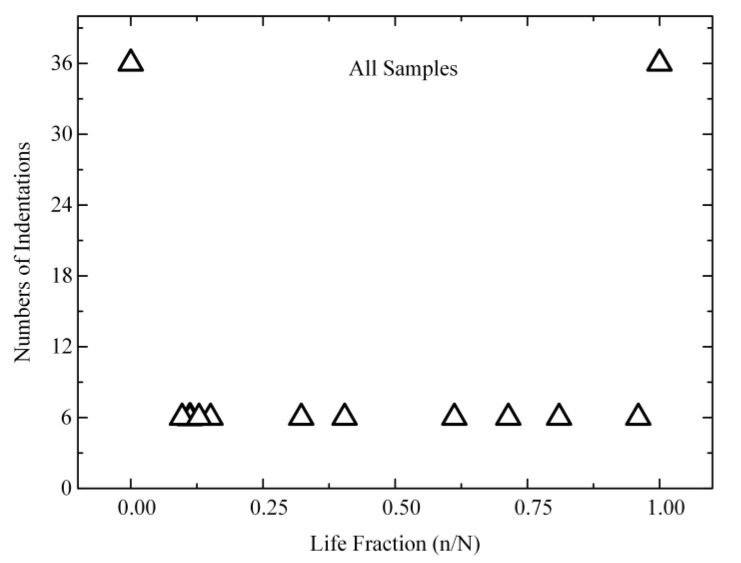
A graphic of the relationship between the nanoindentation test and fatigue life.

Four tests were performed on the four of all six samples and 6 indents were pressed in every test. Figure 1a illustrated where the indents were placed in each of the four time periods. The space of each indentation was 20 µm. The indentation position of each tests was at 20 µm, which also validated the comparability between each test. The specimen underwent 15,000 fatigue loadings before the second indentation test and then underwent 80,000 fatigue loadings before the third indentation test. The last indentation test was taken after fracture. Three tests were performed on the two samples. The experiment successfully captured all four characteristic phases of bone damage: (I) non-fatigue; (II) the early period of fatigue damage (10–40% of the fatigue life); (III) the late period of fatigue damage (60–96% of the fatigue life); and (IV) after fracture. The tests’ complete details are presented in Figure 1 and Figure 2.

As the geometrical properties of specimens have changed significantly after fracture, most mechanical experiments cannot be done effectively. Thus, the nanoindentation test was used to capture the post-fracture information. Nanoindentation has become one of the most important methods to determine the mechanical properties of bone at microscale. The load-displacement curves obtained from nanoindentation can help explain how the bone’s ability to resist fracture is altered by fatigue-induced changes in bone architecture. The sample modulus (*E_samples_*) could be calculated using the Oliver–Pharr method as follows:

(1) $$\frac{1}{E_{r}}=\frac{1v_{sample}^{2}}{E_{sample}}+\frac{1v_{indenter}^{2}}{E_{indenter}}$$

(2) $$E_{r}=\frac{S\sqrt{\pi }}{2\sqrt{A}}$$

where *υ* is Poisson’s ratio, *E_indenter_* = 1140 GPa, *υ_indenter_* = 0.07 (the manual of the NANO G200). S is the unloading slope at the maximum loading.

### 2.3. Statistics

SPSS (IBM SPSS Statistics 26) [50] was used to perform the statistical analysis and Origin 9.0 [51] was used for data fitting. The value of each indentation measurement was used in the statistical analyses. The result of each test is the average of the six indentation values. The standard deviation was also deduced from the corresponding six indentation value. The coefficient of variation (CV%) was calculated for each sample and the maximum value were reported in Table 1. We normalized the modulus of each sample and set the initial modulus as 1, so that the results of all samples could be placed in the same coordinate system. The algorithm used for the nonlinear fitting is the piecewise linear function with two segments in the Origin.

## 3. Results

### 3.1. Optical Microscope and Scanning Electron Microscope (SEM) Analysis 

**Figure 3:**
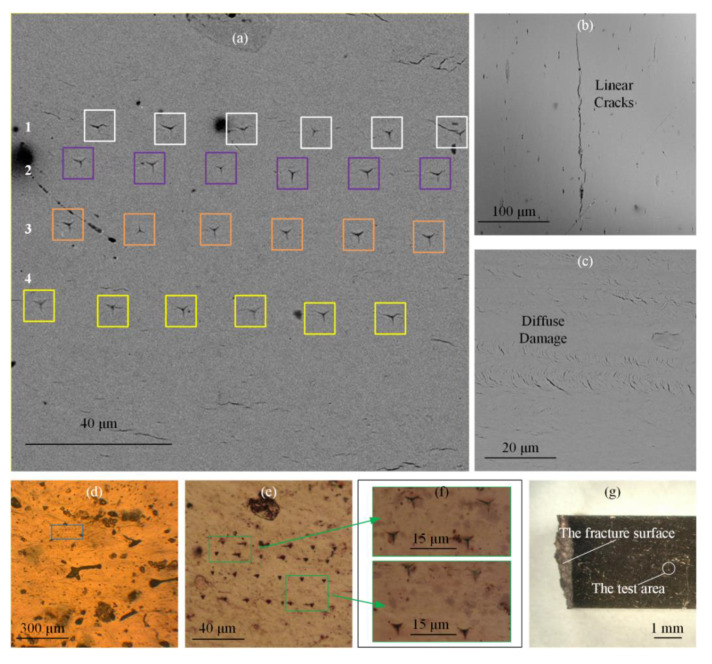
The residual indentations and fatigue damage under optical microscope and SEM. (**a**) SEM image of the indentations; (**b**) Linear cracks; (**c**) Diffuse damage; (**d**–**f**) Optical microscope images of the indentations; (**g**) The fracture surface and the test area. Compared with the damage due to fatigue loading, the damage caused by indentations could be ignored.

The optical microscope Olympus BX51M (Tokyo, Japan) and SEM Phenom XL (Eindhoven, The Netherlands) were used for morphology analysis. Bone samples were spayed with gold using a SBC-12 carbon coater (Beijing, China). This study observed residual indentations on the surface of bone, which undermined bone specimens’ mechanical properties and were a kind of damage themselves. During the entire fatigue loading process, the residual indentation profile was always clear with distinct edges and corners. There were no obvious fatigue cracks originating from the indentations (Figure 3a,e,f). Yin et al. found that the cryo-induced microcracks in bones were not propagated further during micro-indentation testing [52]. The loading used by Yin et al. was 0.245 N~9.8 N, while the loading in this study was about 10 mN. This indicates that the nano-indentation test had little effects on the fatigue resistance of bones. Further, various pores and typical fatigue damage were also visible on the specimens’ surfaces (Figure 3d). Figure 3 shows that scale of pores (>10 μm), linear cracks (100 μm), and diffuse damage (10 μm) was much larger than the residual indentations (4 μm) [13,16,26,27]. In contrast to the structural deterioration caused by fatigue damage, the indentation test could be considered nondestructive. As shown in Figure 3f, the indentation area was several millimeters away from the main failure crack.

### 3.2. Evolution of Reduced Modulus

**Figure 4:**
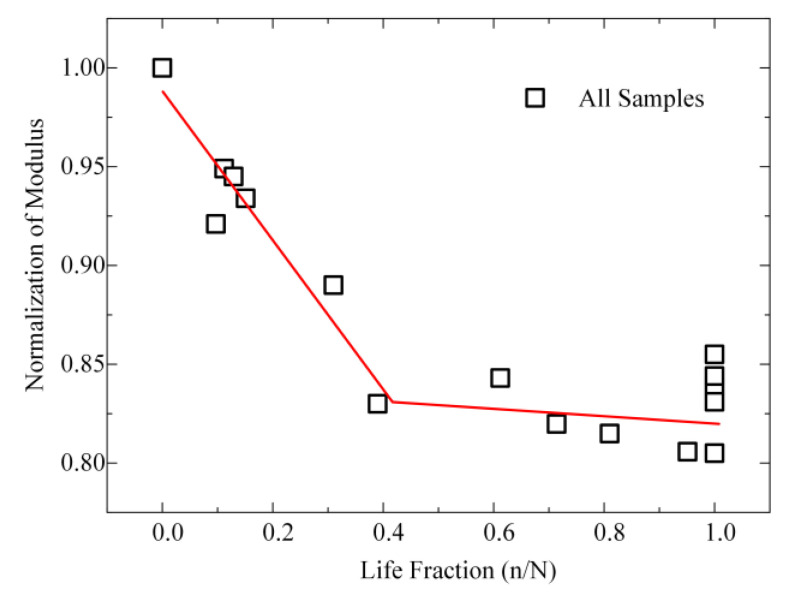
Normalization of the reduced modulus and loading cycles.

The development of fatigue damage in bones is commonly characterized by a reduction in the elastic modulus [6,10,11]. Most studies investigated the reduced modulus of bones in nanoindentation test [27,29,53]. In this study, the value (average ± standard deviation) of the reduced modulus was used in all of the results (Table 1). The reduced modulus of each specimen was normalized, and the initial modulus was set as 1. Fatigue life was used as the life fraction. Figure 4 displays all samples’ results (normalization). The two phases of the reduced modulus are linear with respect to *r*^2^ = 0.89:

(3) $$y=\{\begin{array}{c} 1.0-0.378x,0\leq x\leq 0.42\\ 0.832-0.05\left(x-0.42\right),0.42\leq x\leq 1 \end{array},\;r^{2}=0.89$$

where *y* represents the reduced modulus that is normalized and *x* represents the life fraction, *r* represents the Pearson correlation coefficient.

**Figure 5:**
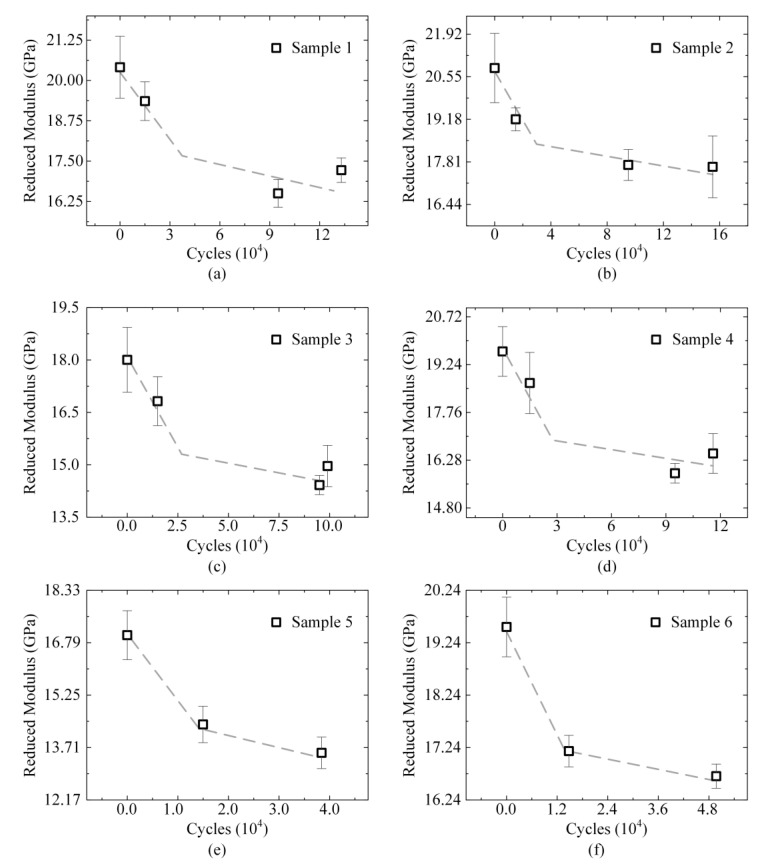
Evolutions of the reduced modulus in the adjacent region of each sample under four-point loading. (**a**–**d**) Reduced modulus of the samples subjected to four times of indentation tests; (**e**,**f**) Reduced modulus of the samples subjected to three times of indentation tests.

The reduced modulus decreased by 17% during the first 40% of the life fraction, after that the variation of the modulus was small until fracture, with a slope of 0.005. As shown in Figure 4 and Figure 5, the development of microscopic damage in the present fatigue tests was non-linear with cycles and followed clearly different trends for the early period and late period of fracture. This non-linear mechanical behavior also was found in many macroscopic tests [6,10,11,27,29,53]. At the late period of fracture, the reduced modulus did not change significantly with cycles. The variations in data were largely from the samples themselves (heterogeneity). It is determined in this study that fatigue loading caused a reduction in the microscopic modulus of bones. This decrease was evident before 40% of the life fraction.

## 4. Discussion

The evolution of bone’s microscopic modulus with fatigue damage was determined in this study. We found that the microscopic modulus of bones decreased largely before 40% of the life fraction whereas proceeded slowly after that. It is interesting to compare these results with those obtained by other researchers who measured the change in modulus on bulk specimens. In the bulk case, the modulus will be affected by cracking as well as by the small-scale cracks. The bone shows a decrease in macroscopic modulus as well as an increase in strain with cycles [6,11,53]. The microcracks accumulation history is commonly divided into three phases during the fatigue damage process, including initiation, accumulation, and failure [10,27,29]. Burr et al. found that significant stained cracks were absent before the loss of modulus reached 15% [29]. Our results show that the microscopic modulus of bone decreased by 17% during the whole cycle loading. In the macroscopic test, the change in modulus was not obvious during the second phase of damage accumulation [10]. The variation of microscopic modulus was also small at the accumulation stage in this study. However, the existing cracks continued to grow during the second period while the accumulation rate of small-scale cracks (<1 μm) was considered to decrease. This suggests indirectly that the larger cracks may be bad at decreasing the elastic modulus of bones. These cracks should be good at releasing higher local stress. 

It is well known that bones are strong and tough because both the intrinsic and extrinsic toughening mechanisms contribute to its ability to resist damage [15,24,31,33]. The intrinsic toughening mechanisms (<1 μm) consists of intrafibrillar toughening, interfibrillar sliding and sacrificial bonding, which operate ahead of the tip of a crack [30]. The extrinsic toughening mechanisms (1~100 μm) includes constrained microcracking, crack bridging, crack deflection and twist, which have no effect on the inherent fracture resistance of the material and act mostly behind the crack tip [15,31]. The internal toughening mechanisms predominate at the early stage of fatigue damage. As the cracks propagate with damage accumulation, constrained microcracking starts to act. The external toughening mechanisms at larger scales predominates when some cracks reach hundreds of micrometers [20,27,30,32,45]. Crack deflection and bridging are also the major contributors to bone toughness [15,30,31]. The dimensions of the residual indentations are 4 μm × 1 μm (width × depth) while both the intrinsic and extrinsic toughening mechanisms can operate in the measured area. The weakening process of the microscopic modulus probably reflects the gradual change from the dominance of intrinsic mechanisms to that of extrinsic mechanisms. This phenomenon is worth investigating further.

Our results demonstrated that the microscopic modulus weakened rapidly at the early period of fracture. Although fatigue damage continued to accumulate at the microscale, its effect on the modulus was limited during the second period. Bones are strongly anisotropic [54,55]. Fan et al. found that the indentation modulus was related to the indentation direction [56]. Our study’s results actually reflected the weakening of the modulus, under the condition of that the direction of the pressed loading was perpendicular to the longitudinal direction (Figure 1c). Fatigue damage will weaken the shear between fibers. This degradation may impact the mechanical properties in the direction that is parallel to the fibers. During 40~100% of the life fraction, the effects of fatigue damage on the micromechanical properties may be manifested in other directions. 

The coefficient of variation (CV%) was calculated at different degrees of damage. The maximum value for each sample is shown in Table 1. Isaksson et al. reported that the CV of reduced modulus in cortical bones was 17% [57]. This value is much higher than what we found. Since the tested area in our study was the bone matrix, which had a high degree of mineralization, the variation range of indentation position was relatively small (100 μm). This setting largely avoided the effects of bone inhomogeneity. The present values of the reduced modulus are similar to those reported previously [56,57,58]. Tai et al. found that the reduced modulus in bone samples from various animals ranged from ~2GPa to 30 GPa [45]. Our results in bovine tibia were within this range and further support this study’s findings.

The fatigue test was performed under the same parameter of loadings. The higher the loading stress, the smaller the cycle life [21]. As the cracks are easy to grow under the high stress, and damage at the smaller scales may not have sufficient time to accumulate. It is expected that the decrease of modulus is small under low cycle fatigue. The exact results require further investigation. The samples were taken from two 18-month-old cattle. Age, disease, and different individuals will affect the properties of samples, so multiple specimens may lead to scattered data [9,13,39]. All samples were dehydrated. Dry bones used in many studies contribute to controlling the experimental environment. Young’s modulus (*E*) is lower by 30–50% in hydrated bone samples compared to the dehydrated ones. It is expected that the dehydration stiffens the bone by altering the viscoelastic properties of collagen. However, drying has no effect on the comparative trends between the samples. The consideration of freshness effect requires a dedicated study, which is outside the scope of this study.

Perturbations in the nano/microstructure of bones can affect its damage tolerance [30]. With the weakening of micromechanical properties, microcracks tend to originate from the sites with higher mineralization, which this study focused on [59,60,61]. The size of linear cracks is comparable to that of the Haversian system, the main structure in a compact bone. They make up the two important toughening mechanisms in bones (i.e., crack deflection and bridging). Diffuse damage can also have a self-locking effect, which is easy to form but difficult to grow. Linear microcracks do not evolve from diffuse damage, they are two unique types of damage. It is still unclear whether the main crack that cause macroscopic failure originates from the existing large cracks in bones or appears suddenly and then propagates rapidly. There are many tiny microcracks dispersing throughout the bones. They are in close proximity, but obviously isolated from each other under static loadings [28,53]. The interaction between them under fatigue loading is still unclear. The mechanisms and events preceding the macroscopic failure of bones require further studies.

## 5. Conclusions

Bone strength and ductility originate at the submicrometer and bone toughness is mostly generated at hundreds of micrometers. However, most fatigue tests have been limited to the macroscale and there is a need to conduct fatigue tests at the microscale. The evolution of bone’s micromechanical properties with fatigue damage was explored in this paper. Nanoindentation tests were conducted at different degrees of damage, including fracture. As the size of the residual indentations was at about 4 μm in length, the degradation of bone’s reduced modulus derived from fatigue damage manifesting at submicrometers. The results showed that bone’s reduced modulus decreased significantly during the initial 40% of the life fraction. However, the variation of reduced modulus was small with cycles after that. The exact mechanism for that cannot be determined in the current study and is worth further investigation.

## Figures and Tables

**Table 1:** Fatigue life, reduced modulus and CV% of the samples.

Samples	Cycles (10^4^)	Modulus (0)	Modulus (1.5)	Modulus (9.5)	Modulus (Failure)	CV_max_
1	13.3	20.41 ± 0.94	19.36 ± 0.60	16.70 ± 0.43	17.22 ± 0.38	4.61%
2	15.5	20.83 ± 1.12	19.18 ± 0.37	17.71 ± 0.48	17.62 ± 1.02	5.79%
3	9.9	18.04 ± 1.23	17.05 ± 0.70	14.66 ± 0.28	14.84 ± 0.59	6.82%
4	11.6	19.64 ± 0.77	18.80 ± 0.95	15.93 ± 0.30	16.49 ± 0.62	5.05%
5	3.8	16.95 ± 0.59	14.11 ± 0.55	-	13.66 ± 0.52	3.81%
6	4.9	19.57 ± 0.85	17.53 ± 0.48	-	16.74 ± 0.33	4.34%

Modulus = reduced modulus (GPa); the numbers in brackets represent loading cycles (10^4^).

## Data Availability

The raw/processed data required to reproduce these findings cannot be shared at this time as the data also forms part of an ongoing study.
